# Two Novel Compound Heterozygous Mutations in the TRAPPC9 Gene Reveal a Connection of Non-syndromic Intellectual Disability and Autism Spectrum Disorder

**DOI:** 10.3389/fgene.2020.00972

**Published:** 2021-02-25

**Authors:** Johannes Krämer, Meinrad Beer, Harald Bode, Benedikt Winter

**Affiliations:** ^1^Division of Pediatric Neurology and Inborn Errors of Metabolism, Children’s Hospital, Ulm University, Ulm, Germany; ^2^Department of Radiology, Ulm University, Ulm, Germany

**Keywords:** autism, TRAPPC9, intellectual disability, genetics, pediatrics

## Abstract

**Introduction:**

Autism spectrum disorder (ASD) is characterized by deficits in communication, social interaction, and repetitive behavior. Up to 70% of ASD cases are linked with intellectual disability (ID). The major genetic causes for ASD and ID are largely unknown, however, a shared genetic etiology between ASD and ID must be assumed. The trafficking protein particle complex subunit 9 (TRAPPC9) is highly expressed in postmitotic neurons of the cerebral cortex, playing a key role in development. Among 43 reported cases with mutations in TRAPPC9, all (100%) showed ID and developmental delay. Among the cases including information about ASD, 26% were affected (19 cases with information, among them 5 with ASD). Nevertheless, in some cases not classified as ASD, descriptions of autistic features like hand-flapping movements were present.

**Clinical Findings:**

The affected individual presented with delay of speech development. Physical development was normal. Besides lateral slope of the eye-lid axis no facial abnormalities were evident. The individual was diagnosed with ID and ASD by structured testing. Cerebral MRI revealed associated abnormalities.

**Genetical Findings:**

The chromosome set was 46,XY without structural changes. Array-CGH showed a normal molecular karyotype (arr(1-22)x2,(X,Y)x1). PCR for the FMR1 gene showed 41 ± 1 CGG repeats, and therefore no evidence of fragile X syndrome. A panel diagnostic for syndromal ID (CASK, EP300, HIVEP2, KIF1A, TRAPPC9) revealed two structural changes in TRAPPC9 in the compound heterozygosity. The mutations c.1678C > T (p.Arg560Cys) and c.3370C > T (p.Pro1124Ser) are classified as missense mutations and are both not described in the literature.

**Conclusion:**

We report two new missense mutations in the TRAPPC9 gene in one individual with ID and ASD. The TRAPPC9 gene should be part of the diagnostic assessment in ID. ASD must be considered as a feature of TRAPPC9-associated ID. It might have been neglected in the literature and should result in specific testing for ASD in affected individuals.

## Introduction

Autism spectrum disorder (ASD) is characterized by deficits in communication, social interaction, and repetitive behavior. Up to 70% of ASD cases are linked with intellectual disability (ID) (Yeargin-Allsopp et al., 2003; Chakrabarti and Fombonne, 2005; Christensen et al., 2016). ID is characterized by an intelligence quotient (IQ) < 70 with onset in childhood, accompanied by deficits in adaptive behaviors that interfere with everyday life. ID affects more than 1% of children worldwide. About an estimated 25% of the cases of all individuals with non-syndromic forms are linked to autosomal recessive disorders and are particularly prevalent in genetic isolates and highly consanguineous populations (Roeleveld et al., 1997). The major genetic causes for ASD and ID are largely unknown, however, a shared genetic etiology between ASD and ID must be assumed, affecting genes in brain development, functions, and neuronal pathways, and also in the enteric nervous system (Bodnar et al., 2020). Genetic heterogeneity and its usual non-syndromic nature make it difficult to achieve consistent genotype–phenotype correlations. Homozygous mutations in the TRAPPC9 gene are reported to be linked to brain abnormalities, microcephaly, ID, developmental delay (Mir et al., 2009; Mochida et al., 2009; Philippe et al., 2009; Koifman et al., 2010; Abou Jamra et al., 2011; Kakar et al., 2012; Marangi et al., 2013; Giorgio et al., 2016; Abbasi et al., 2017), and is thus in combination with ASD (Mortreux et al., 2018; Hnoonual et al., 2002).

The trafficking protein particle complex subunit 9 (TRAPPC9) is highly expressed in postmitotic neurons of the cerebral cortex (Mochida et al., 2009) and is responsible for the activation of nuclear factor kappa b (NFκB) and intracellular trafficking as well as the interaction with the inhibitor of nuclear factor kappa-B kinase (IκK-beta) and thus with the NFκB-kinase (NIK) (Hu et al., 2005). NFκB is a ubiquitously expressed molecule that regulates the expression of a variety of genes, playing a key role in development. This pathway is known to be involved in other diseases with ID, such as autosomal recessive mental retardation 3 (MRT 3, MM#608443) (Basel-Vanagaite et al., 2006) and MRT 13 (#MM613192). TRAPPC9 is also involved in sorting and recycling proteins from the plasma membrane (Cai et al., 2005) as a part of the TRAPPII trafficking pathway (Sacher et al., 2008).

Among the 43 reported cases with mutations in TRAPPC9 in the literature all (100%) individuals showed ID and developmental delay. ASD was reported in 5 cases (12%), whereupon no information about specific symptoms for ASD or structured testing was available in 47% of the reports (20 cases). Among the cases including information about ASD, 26% were affected (19 cases with information, among them 5 with ASD). Nevertheless, in some cases not classified as ASD, descriptions of autistic features like hand-flapping movements were present. Most families were from Persian origin (41 cases, 95%), two cases were reported in Caucasian families (5%).

In this report we firstly describe two new compound heterozygous variants in the TRAPPC9 gene in a male Caucasian patient with ID, ASD, and brain abnormalities.

## Materials and Methods

One male individual with developmental delay and typical symptoms for ASD was ascertained by diagnostic genetic testing to determine the genetic risk for future children of the parents. Blood samples were taken from the child and his parents, DNA was extracted using standard laboratory protocols: target enrichment (TruSight One Expanded) and following NGS (MiSeq, NextSeq Illumina) of the coding areas of interest. An analysis of the sequences was performed with SeqNext^®^ (JSI) and GSvar^®^ by comparing the individual sequence with the given information in the database. Intronic splice site mutations more than 10 bp away from the exon/intron transition were unlikely to be captured by this method. The identified mutations were verified through Sanger sequencing and segregation analysis.

## Results

### Clinical Findings

**FIGURE 1:**
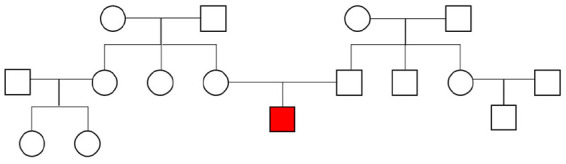
Family pedigree. The affected individual is marked in red.

The affected individual presented with a delay of speech development at the age of 3 years and 5 months, physical development was normal with 17 kg in body weight (75th centile), a height of 101 cm (75th centile), and a head circumference of 51 cm (25th centile). He was able to sit at 9 months and started walking at 15 months. First words were used at the age of 2 years. At 3 years he was able to use three words, but difficulties in receptive language were also evident. There was no history of seizures. Besides lateral slope of the eye-lid axis no facial abnormalities were evident. The interaction was dominated by a total lack of eye-contact and other autistic symptoms like hand-flapping movements and mechanistic body contact to parents and the investigator. Family history revealed no other cases of developmental delay or ASD. Both parents had reached normal school graduation, the father graduated in civil engineering, the mother had completed her education as a nurse. The family pedigree is presented in Figure 1. At the age of 4 years and 5 months the individual still showed no directed use of language but furthermore normal motor development was seen. Potty training was still unsuccessful. Hand-flapping movements, irregular jumping in context of emotion, and non-communicative noises were also evident. Physical development was also still normal with 18.9 kg in body weight (75th centile), a height of 110 cm (75th centile), and a head circumference of 53 cm (50th centile). The individual was diagnosed with ASD according to the autism diagnostic observation schedule 2 (ADOS-2): social affects subscale 20 points, subscale restrictive and repetitive behavior 6 points, total 26 points (cut-off value 16 points, 25–26 points for age 3 years equivalent to symptom score 10 out of 10). Autism-diagnostic interview (ADI-R): subscale A, abnormality in reciprocal interaction 23 points (cut-off 10 points), subscale B, abnormality in communication 14 points (cut-off 8 points), subscale C, repetitive, restrictive, and stereotype behavior 10 points (cut-off 3 points), subscale D, abnormal development until 36 months 3 points (cut-off 1 point), and the German version of the Social Communication Questionnaire (SCQ) total values: completed by parents 21 points (cut-off 16 points), completed by a kindergarten teacher 21 points (cut-off 16 points), completed by a remedial teacher 21 points (cut-off 16 points).

Auditory findings, including brain-stem evoked response audiometry (BERA, threshold level 10 db) and transitory evoked otoacoustic emissions (TEOAE) were both normal.

### Cerebral MRI

**FIGURE 2:**
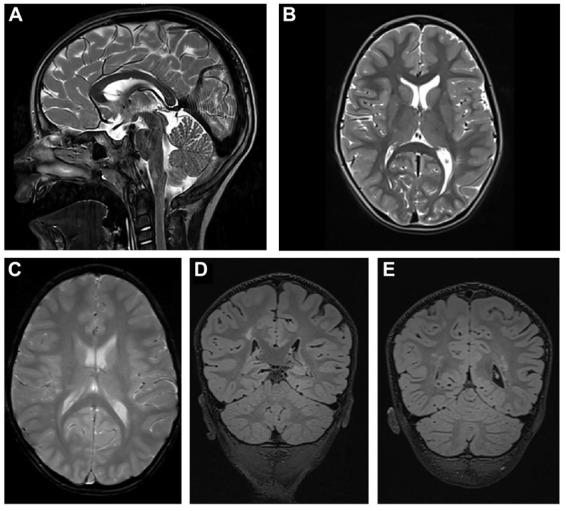
Cerebral MRI. **(A)** T2-weighted sagittal TSE image of the patient shows a slightly thinned but fully formed corpus callosum and normal cerebellar structures. **(B)** T2-weighted axial TSE image of the patient reveals normal gyral pattern of the cerebral cortex and normal volume of white matter but delayed myelination of the internal capsules. **(C)** T2-weighted axial GRE image (T2^∗^) shows diffuse white matter abnormalities in the periventricular and subcortical. **(D,E)** T2-weighted coronal 3D-FLAIR images demonstrate focal as well as diffuse areas of hyperintensity in periventricular white matter.

The cerebral MRI (Figure 2) at the age of 4 years and 8 months showed a thinned but fully formed corpus callosum and normal cerebellar structures. A normal gyral pattern and normal volume of white matter was evident besides delayed myelination of the internal capsules. Abnormal circumscribed areas of T2W hyperintensities in periventricular white matter were evident besides diffuse white matter abnormalities in the periventricular and subcortical.

### Genetical Findings

**FIGURE 3:**
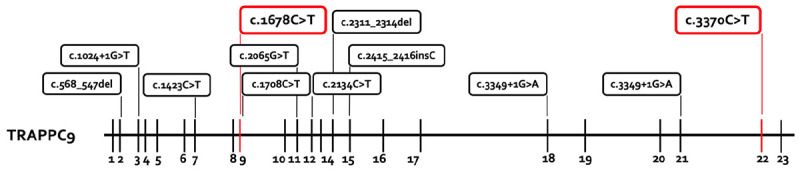
Summary of the reported TRAPPC9 mutations. All mutations previously reported are presented in the box with black lines. The new mutations from this report are marked in the box with red lines. The mutations c.568_574del and c.2134C > T are only reported in the compound heterozygosity with the deletion variants of 8q24.3.

The chromosome set was 46,XY without structural changes. Array-CGH showed a normal molecular karyotype (arr(1-22)x2,(X,Y)x1). PCR for the FMR1 gene showed 41 ± 1 CGG repeats, and therefore there was no evidence of fragile X syndrome. A panel diagnostic for syndromal ID (CASK, EP300, HIVEP2, KIF1A, TRAPPC9) revealed two structural changes in TRAPPC9 in the compound heterozygosity. The mutations c.1678C > T (p.Arg560Cys, Exon 9) and c.3370C > T (p.Pro1124Ser, Exon 22) are classified as missense mutations and are both not described in literature. A summary of the reported TRAPPC9 mutations is given in Figure 3. The genetic analysis of the mother showed the c.1678C > T mutation, the analysis of the father revealed the c.3370C > T mutation in TRAPPC9, thus proving the compound heterozygosity in the affected individual.

## Discussion

Trafficking protein particle complex subunit 9 gene codes for the trafficking protein particle complex 9 (MIM 61699) contains 23 exons and is expressed in muscle, kidney, heart, placenta, and brain (postmitotic neurons) (Hu et al., 2005; Mochida et al., 2009). The protein interacts directly with NIK and IκK-beta, which are part of the NFκB pathways involved in neuronal cell differentiation, synaptic plasticity, neurogenesis (Denis-Donini et al., 2008), and myelination (Philippe et al., 2013; Blank and Prinz, 2014). In the argumentation, TRAPPC9 defects that lead to clinical manifestations of ID and ASD were traceable.

To our knowledge, ten different mutations in the TRAPPC9 gene have been reported in 43 different patients in 16 pedigrees, mainly in homozygous conditions in consanguine families from Persian origin. Only three reports revealed compound heterozygous conditions in an Italian, a French and a Thai pedigree. In this study we can support the previous findings and add two novel missense mutations to the genetic pool for autosomal recessive ID. The clinical presentation of all 44 patients is summarized in Table 1. All patients showed ID and developmental delay in childhood. Secondary microcephaly was described in 95% of the cases in literature, but not in our case. Considering that two cases in the literature with normal head circumferences and our case were diagnosed in childhood [3 years (this case), 10 years (Mortreux et al., 2018), and 14 years (Mir et al., 2009)], the development of secondary microcephaly might be a feature of adolescence and consecutively results in microcephaly only in adulthood. This might also be the case for obesity, which was reported in 47% of the literature. Additionally, it is remarkable that the appearance of speech disorders in all the cases with TRAPPC9 mutations and the high prevalence of autistic features (26%) in cases with specific information about ASD or structured testing (19 cases), besides some cases not being classified as ASD but including descriptions of autistic features like hand-flapping movements. Among the reported cases, a correlation of genetical findings and the appearance of autistic features (genotype-phenotype correlation) was not evident. Additionally, it is remarkable that in the presented case the cerebral MRI not only showed mild cerebral abnormalities but also abnormalities of the white matter as repeatedly reported in the literature. Also the mild facial abnormalities suggest a connection to milder impairment of individuals affected by the reported mutations. Dysmorphic facial features are described in 56% of the cases and thus it seems to be a common, but not obligatory clinical feature.

**TABLE 1:** Patients with homozygous/compound heterozygous mutation or deletion/duplication in TRAPPC9.

	**No ASD (*n* = 18)**	**ASD (*n* = 5)**	**No information about ASD (*n* = 20)**	**This study (*n* = 1)**	**Total reports**
Origin	Pakistan, Arab, Italian, Algerian	Tunisian, Italian, French, Thai	Pakistan, Iranian, Tunisian, Filipino, Syrian, Egyptian	German	44 (100%)
Autistic features	0	5	n/a	1	6/24 (25%)
Age at report	15.9 ± 13.5	8.2 ± 2.5	17.1 ± 10.2	n/a	14.9 ± 11.4
Male/Female	2:16	2:3	14:6	1:0	19:25
Intellectual disability	18	5	20	1	44/44 (100%)
Developmental delay	18	5	20	1	44/44 (100%)
Microcephaly	16	4	18	1	39/42 (93%)
Obesity	4	3	5	0	12/24 (50%)
Seizure	2	0	3	0	5/32 (16%)
Hand-flapping movements	8	n/a	1	1	10/13 (77%)
Brain abnormalities	10	5	2	1	18/18 (100%)
Dysmorphic face	5	4	11	0	20/37 (54%)

The individual in the current study and reported other individuals with an identification of TRAPPC9 mutations in childhood and diagnosis of ASD reveal ASD as a feature of TRAPPC9-associated ID which might have been neglected in previous literature and should result in closer examination in future reports.

## Conclusion

We report, to our knowledge, two not yet described missense mutations in the TRAPPC9 gene in one individual with ID and ASD. The TRAPPC9 gene should be part of the diagnostic assessment in ID. ASD must be considered as a feature of TRAPPC9-associated ID. It might have been neglected in previous literature and should result in specific testing for ASD in affected individuals.

## Limitations

For a detailed work-up, a family screening should have been performed. Due to long travel distances to the study center no additional family member was examined. For underlining the pathogenetic relevance of the mutation we found, further individuals should be described. At the time of publication, no other individuals with TRAPPC9 mutations were under treatment in our center.

## Data Availability Statement

The datasets for this manuscript are not publicly available because of missing consent of the laboratory. Requests to access the datasets should be directed to the corresponding author.

## Ethics Statement

No human or animal studies are presented. No potentially identifiable human images or data is presented in this study. Thus, no consent is required.

## Author Contributions

JK: interpretation of the data, manuscript, first draft, and revision. MB: interpretation of radiological findings and revision. BW: interpretation of the data and revision. HB: revision. All authors contributed to the article and approved the submitted version.

## Conflict of Interest

The authors declare that the research was conducted in the absence of any commercial or financial relationships that could be construed as a potential conflict of interest.

## Abbreviations

ASD: Autism spectrum disorder
ADOS-2: Autism diagnostic observation schedule 2
BERA: Brain-stem evoked response audiometry
TEOAE: Transitory evoked otoacoustic emissions
CNV: Copy number variations
ID: Intellectual disability
I κ K-beta: Inhibitor of nuclear factor kappa-B kinase
IQ: Intelligence quotient
MRI: Magnetic resonance imaging
NF κ B: Nuclear factor kappa b
NIK: NF κ B-kinase
SCQ: Social Communication Questionnaire
TRAPPC9: Trafficking protein particle complex subunit 9.
